# *GDPD3* Deficiency Alleviates Neuropathic Pain and Reprograms Macrophagic Polarization Through PGE2 and PPARγ Pathway

**DOI:** 10.1007/s11064-024-04148-2

**Published:** 2024-05-20

**Authors:** Wenqian Li, Youjia Fan, Haizhen Lan, Xiaoxiao Li, Qichao Wu, Rong Dong

**Affiliations:** 1 Department of Anesthesiology, Zhongshan Hospital, Fudan University, Shanghai, China; 2 Department of Anesthesiology, Ruijin Hospital, Shanghai Jiao Tong University School of Medicine, 197 Ruijin Second Road, Shanghai, 200025 China

**Keywords:** Peripheral pain, Gdpd3, Macrophages, PPARγ/FABP4 pathway

## Abstract

The complex mechanism of neuropathic pain involves various aspects of both central and peripheral pain conduction pathways. An effective cure for neuropathic pain therefore remains elusive. We found that deficiency of the gene *Gdpd3*, encoding a lysophospholipase D enzyme, alleviates the inflammatory responses in dorsal root ganglia (DRG) of mice under neuropathic pain and reduces PE (20:4) and PGE2 in DRG. *Gdpd3* deficiency had a stronger analgesic effect on neuropathic pain than Celecoxib, a nonsteroidal anti-inflammatory drug. *Gdpd3* deficiency also interferes with the polarization of macrophages, switching from M1 towards M2 phenotype. The PPARγ/ FABP4 pathway was screened by RNA sequencing as functional related with *Gdpd3* deficient BMDMs stimulated with LPS. Both protein and mRNA levels of PPARγ in *GDPD3* deficient BMDMs were higher than those of the litter control mice. However, GW9962 (inhibitor of PPARγ) could reverse the reprogramming polarization of macrophages caused by *GDPD3* deficiency. Therefore, our study suggests that *GDPD3* deficiency exerts a relieving effect on neuropathic pain and alleviates neuroinflammation in DRG by switching the phenotype of macrophages from M1 to M2, which was mediated through PGE2 and PPARγ/ FABP4 pathway.

**Supplementary Information:**

The online version contains supplementary material available at 10.1007/s11064-024-04148-2.

## Introduction

Nearly 10% of the general population suffer from neuropathic pain, which is still hard to cure [1]. Neuroinflammation plays either positive or negative role in the transition to and perpetuation of neuropathic pain [2]. Macrophages play a pivotal role in the resolution of inflammation and regeneration of injured tissues. Resident and infiltrating macrophages, existing in dynamic switching phenotypes depending on environment, are found in both central and peripheral nerve system. The phenotypes of macrophages have different effects on neuroinflammation in DRG [3]. Paralleling the mechanical hypersensitivity after peripheral nerve injury is a significant expansion and proliferation of DRG macrophages. DRG macrophages critically contribute to both the initiation and maintenance of the mechanical hypersensitivity that characterizes the neuropathic pain [4]. Lipid metabolism has recently been introduced to the mechanism of neuropathic pain. Extracellular lipid mediators, signaling via specific G protein-coupled receptors, regulate the development, physiological functions, and pathological processes of various type of cellular stress including neuropathic pain [5]. Phospholipid was found to restrain the proinflammatory M1-like phenotype in human macrophages [6]. Our study aimed to explore the role of glycerophosphodiester phosphodiesterase domain containing 3 (GDPD3) a lysophosphatidylcholine D enzyme also known as glycerophosphodiester phosphodiesterase 7 (GDE7), in modulating macrophagic polarization and neuroinflammation under neuropathic pain.

In this study, we found that after neuropathic pain in CCI mice, macrophages in DRG polarized toward M1, and the expression of GDPD3 increased significantly in DRG. In the in vitro experiment, GDPD3 deficiency was demonstrated to reprogram the polarization of BMDMs and the transcription of inflammatory cytokines. As for pain behavior, GDPD3 deficiency significantly restrained the mechanical and thermal hypersensitivity in mice with neuropathic pain. Celecoxib, the inhibitor of prostaglandin E2 (PGE2), which is therapeutic in the neuropathic pain-induced oxidative stress [7], reversed the promotion effect of GDPD3 on proinflammatory cytokines (IL-1β and TNF-α) in BMDMs challenged with LPS. Pathway analysis of RNA-sequencing highly enriched the PPAR γ/FABP4 pathway to be related with GDPD3 deficient BMDMs challenged with LPS. PPARγ activation was reported to have anti-inflammatory properties by promoting macrophagic polarization shift towards M2 phenotype [8]. There is a complex regulatory relationship between PGE2 and PPAR γ/FABP4 pathway, and in skin inflammatory responses, polymethoxyflavones inhibit PGE2 production via PPARγ [9]; whereas in the chondritis model, knockdown of FABP4 inhibits the expression level of PGE2 [10].

Altogether, these findings suggested that GDPD3 deficiency can not only modulate lipid metabolism but also reprograms macrophagic polarization through PGE2 and PPARγ pathway, thereby alleviating neuropathic pain. This helps to better understand the mechanisms of neuropathic pain and provides a new important potential therapeutic option.

## Materials and Methods

### Generation of Mice

C57BL/6J mice (aged 7–9 weeks, 13–20 g) were purchased from Beijing Vital River Laboratory Animal Technology Co., Ltd. C57BL/6JSmoc-*Gdpd3*^*em1Smoc*^ (*Gdpd3*^−/−^) mice (NM-KO-201158) were purchased from Shanghai Model Organisms Center, Inc. *Gdpd3*^*−/−*^ mice were mated with C57BL/6J mice to get *Gdpd3*^±^ heterozygote mice, which were then mated with each other to obtain *Gdpd3*^+*/*+^, *Gdpd3*^+/−^, and *Gdpd3*^*−/−*^ mice. All mice were bred in specific pathogen-free (SPF) conditions. All animal experiments were performed according to the National Institute of Health Guide for the Care and Use of Laboratory Animals, with approval from the Scientific Investigation Board of Jiaotong University School of Medicine, Shanghai, China (ethical code 20230427-02).

### Reagents

LPS from Escherichia coli O111:B4 and IL-4 from mice (SRP3211) were purchased from Sigma-Aldrich. Anti-GDPD3 (ab214375) was purchased from Abcam. Antibodies againstβ-actin (HRP-conjugated) were from Proteinteck. Antibodies against CD86 (E5W6H), CD86/B7-2 (GL-1), CD206/MRC1 (E6T5J), PPARγ (81B8) were purchased from Cell Signaling Technology.

### Chronic Constriction Injury (CCI) Model in Mouse

Animals were anesthetized with 1% sodium pentobarbital (60 mg/kg, i.p.), and the left sciatic nerve was exposed at the middle thigh. 6-0 Prolene sutures was used to make three ligatures loosely around the nerve at the proximal end of the trifurcation, with 1 mm distance between each ligature. A brief twitch at hind limbs was observed. In the sham operation group, same anesthesia and sciatic nerve exposure were performed, but without nerve ligation. The L4-L6 DRGs were removed.

### AAV Injection

The mice were fixed in the prone position on the modified stereotaxic apparatus, and the L4–L6 vertebrae were exposed after disinfection. Under a 10 ×  microscope, a syringe was slowly inserted into the dorsal root nodule at 100 μm, left for 2 min, and then 1 μl of AAV-shRNA-GDPD3 or AAV-shRNA-NC (Hanbio Technology Shanghai, China) was slowly injected, left for 5 min, and then slowly withdrawn from the syringe, and then the dorsal root nodule injections were completed for L4-L6 in turn. In this study, AAV injections were completed 3 weeks prior to CCI modelling to obtain a stable knockout effect.

### Pain Behavior Measurement

Mechanical pain behavior was measured by a calibrated paw withdrawal response to von Frey fibers (Stoelting Co., Wood Dale, IL. USA). Briefly, each mouse was placed in a plexiglass chamber sitting on the elevated mesh net. Low force (0.07 *g*) or high force (0.4 g) von Frey filament was applied to the hind paws for about 1 s, and each stimulation was repeated 10 times. The occurrence of paw withdrawal in each trial was recorded. A percentage response frequency was calculated as (paw withdrawal times/10 trials) × 100. This percentage was used to indicate the withdrawal response.

Thermal pain behavior was measured by Hargreaves test. Briefly, each mouse was placed in a plexiglass box on a glass plate with a light box underneath. The radiant heat generated by Model 336 Pain Relief Apparatus (Science Instruments, Woodland Hills, CA, USA) was applied by pointing a light beam to the middle of the plantar surface of each hind paw. When the animal raised its foot, the beam was turned off. The length of time between the start of the beam and the lifting of the foot is defined as the paw withdrawal latency. Each test was repeated 3 times at 5 min intervals. The maximum of 20 s of heat applying time was set to avoid damage to the hind paw tissue.

### Culture of Bone Marrow-Derived Macrophage (BMDM)

The bone marrow of *Gdpd3*^+/−^ and *Gdpd3*^*−/−*^ mice was collected, mixed with 20 ng/mL M-CSF, and cultured in a 5% CO_2_ incubator for BMDM induction. In later experiments, LPS (50 ng/mL, PeproTech, USA) and IL-4 (10 ng/mL, PeproTech,) were used to stimulate the BMDM for 24 h according to experimental needs.

### RNA-Seq Analysis

NanoDrop ND-2000 (Thermo Scientific) quantitatively analyzed the total RNA of *Gdpd3*^+/−^ and *Gdpd3*^+/−^ BMDMs and detected RNA integrity using Agilent Bioanalyzer 2100 (Agilent Technologies). After the RNA quality inspection is qualified, the labeling of the sample, hybridization of the chip, and elution refer to the chip standard process. The total RNA is firstly reverse transcribed into double stranded cDNA, followed by further synthesis of cRNA labeled with Cyanine-3-CTP (Cy3). The labeled cRNA was hybridized with the chip, and after elution, the original image was scanned using Agilent Scanner G2505C (Agilent Technologies). The Feature Extraction software (version 10.7.1.1, AgilentTechnologies) is used to process the original image and extract the original data, and the GeneSpringGX software (version 14.9, AgilentTechnologies) is used to quantify the original data.

### Reverse Transcription and RT-QPCR

RNA was extracted using Tri-Reagent (Sigma-Aldrich), according to the manufacturer’s instructions. The amount of purified RNA was estimated by a measure of absorbance at 260 nm. Purity was determined as the 260 nm/280 nm OD ratio with expected values between 1.8 and 2.0. Total mRNA (1– 2 μg) was reverse transcribed to cDNA using Reverse Transcription Kit (TaKaRa BIO INC). RT-QPCR experiments were performed on ABI Prism 7500 Real-Time PCR System (Applied Biosystems). In brief, 2 μL of cDNA were used in a RT-QPCR reaction volume of 20 μL, containing 10 μL of SYBR Premix Ex Taq (TaKaRa BIO INC), 0.4 μL of ROX Reference Dye II (50 ×, TaKaRa BIO INC), 0.4 μL forward and 0.4 μL reverse primers and 6.8 μL of water. The reaction conditions were 95 °C for 30 s followed by 40 cycles of 95 °C for 5 s and 60 °C for 34 s. Each sample was quantified for each gene in triplicate and GAPDH was used as a control gene. The primer sequences were shown in Table S1.

### Western Blot Analysis

The samples were homogenized in microfuge tubes with RIPA buffer (Cell Signaling Technology) containing 1 mM PMSF protease inhibitor (Thermo Scientific). The samples were kept on ice for 30 min and centrifuged at 12,000 *g* for 15 min at 4 °C before the lysate supernatants were collected. Protein concentrations of the supernatants were measured by a BCA assay (Pierce) according to the manufacturer’s instructions. The extracts were separated by 12% SDS-PAGE and transferred onto polyvinylidene fluoride membranes. The membranes were blocked by 5% nonfat dried milk in TBS-T (TBS containing 0.1‰ Tween-20) and incubated overnight with the following primary antibodies: anti β-actin (1/1000), anti-GDPD3 (1/1000), PPARγ (1/1000), CD86 (1/2000) and CD206 (1/2000). After incubation with the secondary antibody (peroxidase-conjugated anti-rabbit,1:20,000, Jackson Immuno Research) for 1 h at room temperature, the membranes were scanned. The integrated optical density (IOD) was calculated by use of Odyssey Infrared Imaging System.

### Enzyme-Linked Immunosorbent Assay (ELISA)

The concentration of PGE2 were measured with validated specific ELISA assays according to the manufacturer’s protocol (Elabscience). Briefly, 100 μL of each sample was added to ELISA plates in duplicate. Absorbance was measured on Microplate reader (Thermo Scientific).

### Flow Cytometry

BMDMs were incubated with Zymosan (0.5 mg/mL) or Latex beads (carboxylate-modified polystyrene, fluorescent yellow-green, 1.0 μm) for 1 H.Add corresponding flow cytometry antibodies labeled with fluorescein according to the recommended concentration in the antibody manual, incubate in dark for 30 min (4 °C), wash twice with PBS (4 °C), collect cells, and resuspend them on PBS (400 μL) Medium. Flow cytometry FACSLSR II was used for machine detection.

### Histochemical Staining

The tissue perfused with PBS (4 °C) and 4% Paraformaldehyde sequentially was fixed in 4% Paraformaldehyde. The tissue with the desired plane was cut in the fume hood and placed in the dehydration box. Dehydration box for alcohol gradient dehydration in the dehydrator: 75% alcohol for 4 h; 85% alcohol for 2 h; 90% alcohol for 2 h; 95% alcohol for 1 h; anhydrous ethanol for 30 min × 2; Xylene 10 min × 2; Soak in wax for 1 h × 3. After embedding, place it on a paraffin slicer for slicing (4 μ Lay m/piece flat on a glass slide and dry it in a 60 °C oven. The sections were then dewaxed, rehydrated and the antigen retrieved by high pressure incubation in Tris/EDTA buffer (pH 9.0) for 2 min. The sections were then incubated with 3% hydrogen peroxide for 20 min to inactivate endogenous peroxidase activity, closed with 1% bovine serum albumin for 1 h at room temperature, and incubated with the following primary antibodies: anti-GDPD3 (1/500), anti-CD86 (1/500), and anti-CD206 (1/500). On the following day, sections were incubated with goat anti-rabbit secondary antibody for 60 min at room temperature, washed with TBS, treated with DAB solution, and then observed and imaged with a microscope.

### Mass Spectrometry (MS) Analysis

Lipids were extracted with Solid Phase Extraction (SPE) and QuEChERS (57,171, Merck). The extraction was added to each column: 100% MeOH, 100% H_2_O, sample, 10% MeOH, and 100% MeOH for elution. Samples were dried and taken up in 100 mL buffer A (63% H2O, 37% acetonitrile, 0.02% acetic acid). Five microliters were injected into the ultra-high performance liquid chromatography system. The analysis was performed with a mass spectrometer (6500 Qtrap; Sciex, Framingham, MA, USA). DDA (data-dependent acquisition) mass spectrum techniques were used to acquire tandem MS data on a ThermoFisher Q Exactive mass spectrometer (ThermoFisher, USA) fitted with a Nano Flex ion source. Data were acquired using an ion spray voltage of 1.9 kV, and an interface heater with temperature of 275 °C. For a full mass spectrometry survey scan, the target value was 3 × 10^6 and the scan ranged from 350 to 2000 m/z at a resolution of 70,000 and a maximum injection time of 100 ms.

### Statistical Analysis

Statistical software GraphPad Prism 6.01 (GraphPad Software, Inc.) was used to analyze data. Statistically significant differences between two normally distributed groups were determined using Student’s paired *t*-tests with two-tailed P-values. Differences between three or more groups were determined by one-way ANOVA with Tukey’s multiple comparisons tests. Differences between two or more groups containing more than one variable were determined by two-way ANOVA with Sidak’s multiple comparisons tests. P-values of < 0.05 were considered statistically significant.

## Results

### Establish a Mouse CCI Model for Neuropathic Pain

Compared with mice in the sham operation group, the frequency of foot withdrawal in the CCI group in response to high force (0.4 g) von Frey fibers increased significantly from postoperative day (POD)7 and peaked at POD28 (Fig S1A). The frequencies of foot withdrawal on POD7 (68.3 ± 4.8% Vs 36.7 ± 2.1%), POD14 (86.7 ± 2.1% vs 35.00 ± 4.3%), POD28 (86.7 ± 2.1%Vs 35.00 ± 4.3%), and POD45 (76.7 ± 2.1% vs 35.0 ± 2.2%) were all significantly higher for CCI group than the control group. The foot withdrawal frequency in response to low force (0.07 g) von Frey fibers in the CCI group also increased significantly from POD7 compared to the sham operation group (Fig S1B; 36.67 ± 3.33% vs 18.33 ± 3.07%), and also peaked on POD 21 (46.67 ± 3.33% vs 18.33 ± 3.07%). Consistent with mechanical pain behavior, thermal pain behavior study showed that the thermal pain threshold of the CCI group was significantly lower than that of the sham operation group from POD 7 to POD 28 (Fig S1C). These results indicated that the CCI mouse model established was stable in studying the role of DRG macrophages in neuropathic pain.

### The Macrophage Polarization Expression Trends in Drg During Neuropathic Pain Progressing

**Fig. 1:**
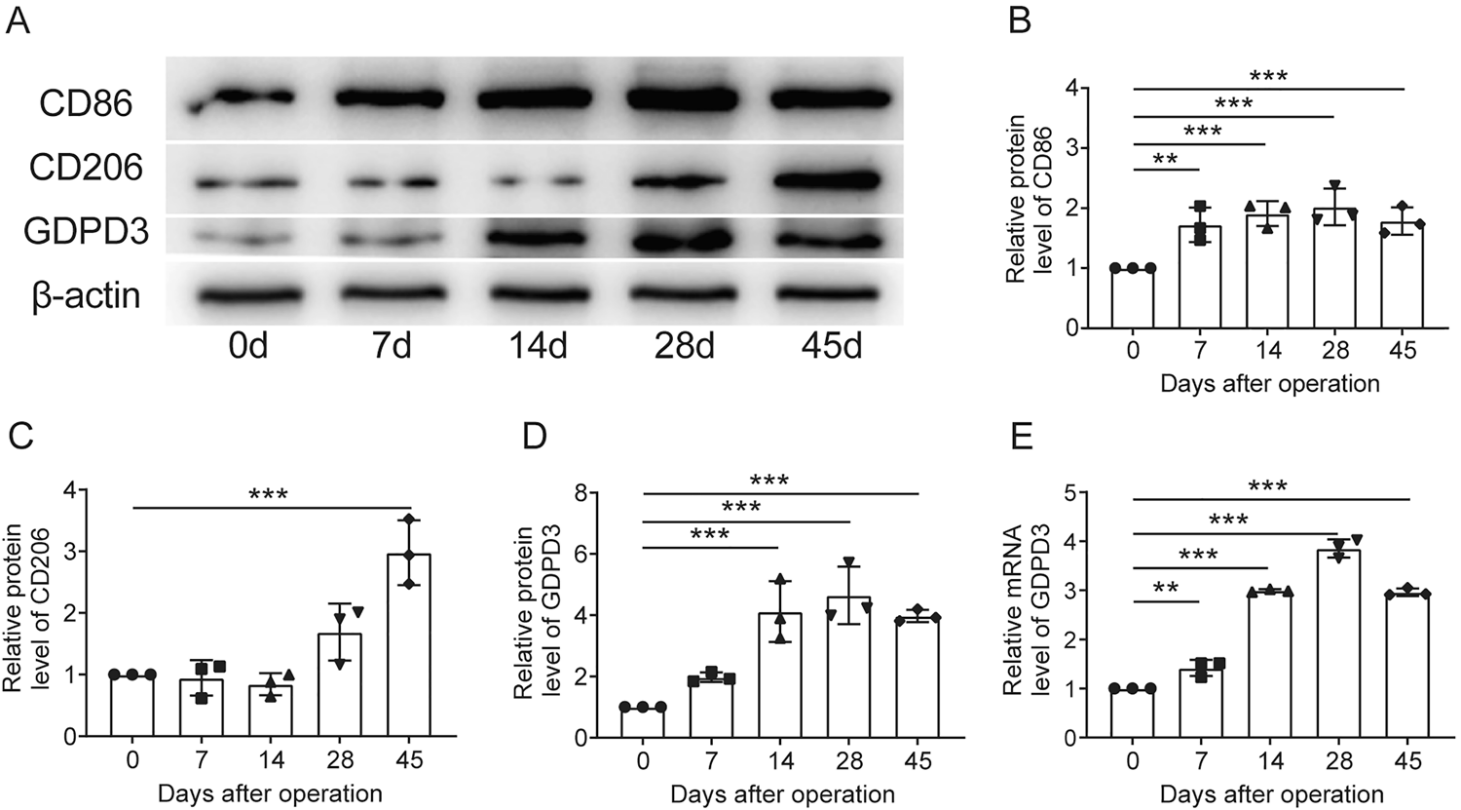
The macrophage polarization and GDPD3 expression trends in DRG during neuropathic pain progressing. **A** Illustration of the Western blot results for macrophage polarization and GDPD3 expression trends in DRG. **B** Statistical trend results of M1 macrophage polarization marker (CD86) tested by Western blot in DRG. **C** Statistical trend results of the trend of M2 macrophage polarization marker (CD206) tested by Western blot in DRG. **D** Statistical GDPD3 relative protein level trends tested by Western blot in DRG. **E** Statistical GDPD3 relative mRNA expression trends tested by RT-QPCR analysis in DRG. The data were presented as mean ± SD, n = 3, **P < 0.01, ***P < 0.001.DRG, dorsal root ganglion

Western blot analysis on the samples obtained from DRGs at various time points after operation was used to determine the polarization of macrophages. We studied the expression of CD86, a classic M1 polarization marker, and CD206, a M2 polarization marker to determine the changes in macrophages located in DRGs. Western blot results were showed in Fig. 1A, while the quantifications of the relative band intensity were shown in Fig.1B, C Compared with the sham group, the CD86 expression of the DRGs in the CCI group increased from POD7 (1.7 ± 0.2 fold) and peaked on POD28 (2.0 ± 0.2 fold) (Fig. 1A, B). On the other hand, expression of CD206 didn’t increase until the POD28 and didn’t become significantly higher than the sham group utill the POD45 (3.0 ± 0.3 fold) (Fig. 1A, B). These results indicated that the increased number of M1-polarization macrophages in the DRGs were consistent with the hyperalgesic behaviour of CCI mice, and the relative deficiency of M2-polarization macrophages might be contributory to neuropathic pain. The mechanisms that influence macrophage polarisation of DRGs in neuropathic pain have interested us

### GDPD3 Expression was Parallel with M1 Polarization of DRG Macrophages

We used western blot and RT-QPCR analysis to assess the expression levels of GDPD3 in DRGs. The protein levels of GDPD3 in DRGs increased from POD7 (2.0 ± 0.1 fold), peaked at day 28 (4.7 ± 0.5fold) (Fig. 1A–D). The changes of mRNA levels of GDPD3 in CCI mice followed a similar pattern but faster in which it became significantly higher than that in the sham group on POD7 (1.4 ± 0.2 fold) and peaked at day 28 (3.9 ± 0.2 fold) (Fig. 1E). Both mRNA and protein levels of GDPD3 in CCI mice fell from the peakon POD45.

**Fig. 2:**
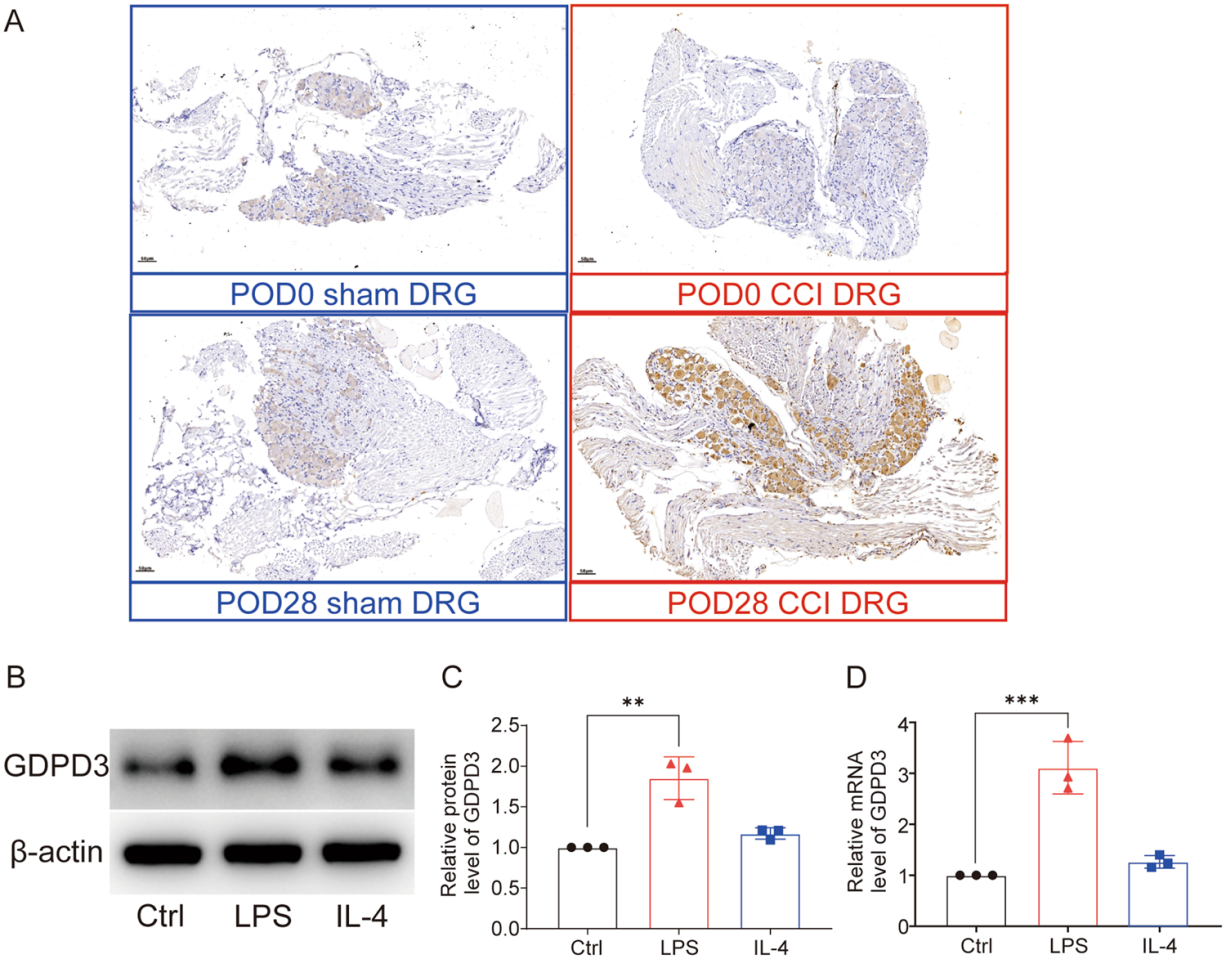
GDPD3 expression in DRG and BMDMs. **A** Comparison of immunohistochemical results of GDPD3 expression trends in DRG between the CCI and sham group. **B**, **C** GDPD3 expression tested by Western blot in M1 polarized BMDMs (LPS 50 ng/ml stimulation) and M2 polarized BMDMs (IL-4 10 ng/ml stimulation). **D** GDPD3 mRNA expression tested by RT-QPCR analysis in M1 polarized and M2 polarized BMDMs. The data were presented as mean ± SD, n = 3, ***P < 0.001. BMDM, bone marrow-derived macrophage; LPS, lipopolysaccharide

Immunohistochemical results showed that the GDPD3 expression of the DRGs in the CCI group was significantly higher than in the sham group on POD28 (Fig. 2A). In vitro, LPS stimulation, which could induce M1 polarization of BMDM, increased the expression of GDPD3 protein and mRNA, while IL4 stimulation (inducing M2 polarization) did not affect the expression of GDPD3 (Fig. 2B–D). It suggested that GDPD3 high expression was parallel with M1 macrophage polarization.

### GDPD3 Deficiency Promoted the Shift From M1 to M2 Polarization of Macrophages

**Fig. 3:**
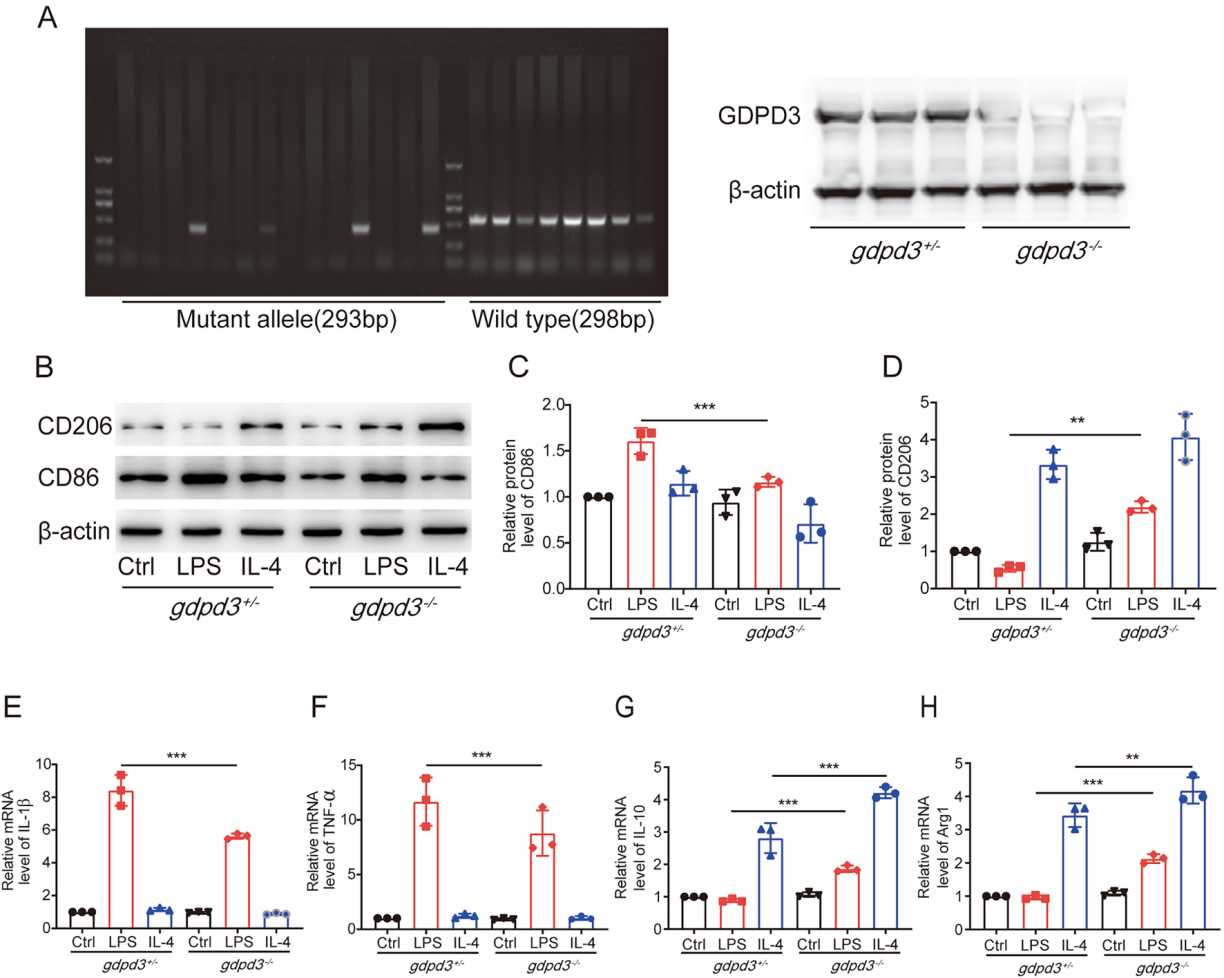
GDPD3 deficiency promotes the transformation of M1 macrophage polarization into M2 in vitro. **A** Gel electrophoresis assay and western blot assay to analyses the expression of GDPD3. **B** Western blot results of M1 macrophage polarization marker CD86 and M2 marker CD206 in GDPD3^−/−^ and GDPD3^+/−^ BMDMs treated with M1 stimulus (LPS) and M2 stimulus (IL-4). **C**, **D** Quantitation of relative band densities of CD86 and CD206 in GDPD3^−/−^ and GDPD3^+/−^ BMDMs treated with LPS and IL-4. **E**, **F** Relative mRNA levels of proinflammatory factor IL-1β and TNF-α in GDPD3^−/−^ and GDPD3^+/−^ BMDMs challenged with LPS and IL-4 tested by RT-QPCR analysis. **G**, **H** Relative M2 macrophage polarization gene IL-10 and Arg1 expression via RT-QPCR analysis in GDPD3^−/−^ and GDPD3^+/−^ BMDMs challenged with LPS and IL-4. The data were presented as mean ± SD, n = 3, **P < 0.01, ***P < 0.001

The role of GDPD3 in macrophageic polarization was further investigated in Gdpd3 deficient (GDPD3^−/−^) BMDMs and their littermate control (GDPD3^+/−^) BMDMs. Before the formal experiments began, the expression levels of GDPD3 in GDPD3^+/−^ and GDPD3^−/−^ mice were observed by gel electrophoresis and western blot to verify the knockdown effect and the reasonableness of GDPD as a control group, and the results showed that there was a significant reduction in the expression levels of GDPD3^−/−^ mice (Fig. 3A). Western blot results showed that GDPD3^−/−^ BMDMs did not show difference in the expression levels of CD86 (M1 marker) or CD206 (M2 marker), comparing to GDPD3^+/−^ BMDMs, suggesting that lack of GDPD3 did not alter the steady state macrophages. When challenged with LPS, the GDPD3^+/−^ BMDMs expressed significantly higher level of CD86, which was not observed in GDPD3^−/−^ BMDMs. The CD86 expression was also reduced in GDPD3^−/−^ BMDMs upon IL-4 treatment in contrast to that in GDPD3^+/−^ BMDMs. The expression of CD206, on the other hand, was significantly increased in both GDPD3^−/−^ and GDPD3^+/−^ BMDMs upon IL-4 treatment (Fig. 3B–D). Interestingly, while the expression of CD206 decreased in GDPD3^+/−^ BMDMs treated with LPS, it increased in GDPD3^−/−^ BMDMs under the same treatment, although without statistical significance.

We then assessed the mRNA levels of polarizing related inflammatory cytokines in those BMDMs under LPS and IL-4 treatment. As shown in Fig. 3E, F, the expressions of M1 genes, IL-1β and TNF-α increased in both GDPD3^−/−^ and GDPD3 ^+/−^ BMDMs treated with LPS. However, the levels of increase in GDPD3^−/−^ BMDMs were relatively lower than those in GDPD3^+/−^ BMDMs. Interestingly, the expression of M2 genes, IL-10 and Arg1, increased in both GDPD3^−/−^ and GDPD3^+/−^BMDMs when treated with either LPS or IL-4 (Fig. 3G, H). Similar to what we observed with CD206, when the BMDMs were treated with LPS or IL-4, the expression of M2 genes increased in GDPD3^−/−^ BMDMs in contrast to GDPD3^+/−^ BMDMs.

### GDPD3 Deficiency Ameliorates CCI-Induced Neuropathic Pain

**Fig. 4:**
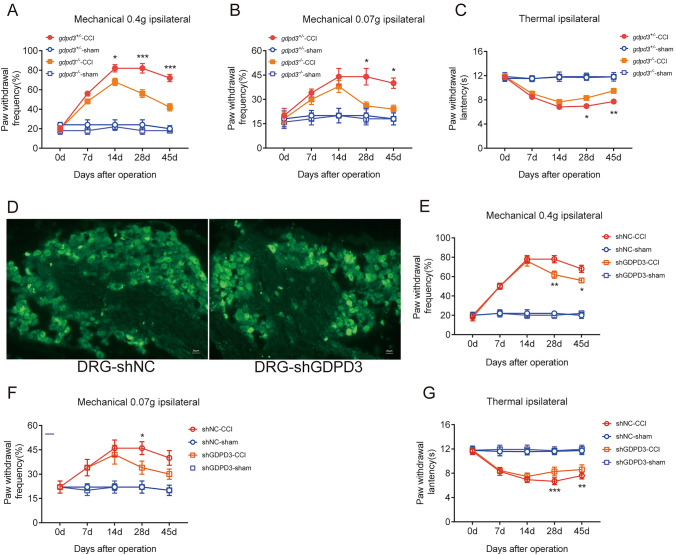
GDPD3 deficiency ameliorates CCI-induced neuropathic pain. GDPD3^−/−^, GDPD3, shNC, and shGDPD3 mice were subjected to the CCI model. Mechanical hypersensitivity trend was assessed by the percentage of paw withdrawal to 0.4 g von Frey filament (**A**, **E**) and 0.07 *g* von Frey filament (**B**, **F**), thermal hypersensitivity trend was assessed by paw withdrawal latency to Hargreaves test (**C**, **G)**. (**D)** Fluorescence microscopy showed virus was injection in the DRG. The data were presented as mean ± SD, n = 3, *P < 0.05, **P < 0.01, ***P < 0.001

In terms of pain behavior, GDPD3^−/−^ mice had lower mechanical and thermal hypersensitivity since CCI POD28 than GDPD3^+/−^ group, indicating that deficiency of GDPD3 could relieve neuropathic pain. The frequency of foot withdrawal in GDPD3^−/−^ mice in response to high force (0.4 g) von Frey fibers was significantly lower than that in GDPD3^+/−^ mice from POD14 and peaked at CCI 28d (Fig. 4A). The foot withdrawal frequency in response to low force (0.07 g) von Frey fibers in GDPD3^−/−^ mice, however, was significantly lower than that in GDPD3^+/−^ mice from POD28 to POD45 (Fig. 4B). The thermal pain threshold of GDPD3^−/−^ mice was higher than that of GDPD3^+/−^ mice from POD28 to POD45 (Fig. 4C). To better demonstrate the role of GDPD3 in neuropathic pain, we silenced GDPD3 gene expression in WT and CCI model mice using AAV localized injection into the DRG (Fig. 4D). Behavioral experiments showed that from CCI POD28 onwards, shRNA-GDPD3 mice were less mechanically and thermally hypersensitive than the WT group, suggesting that GDPD3 deficiency also exerts a role in alleviating neuropathic pain in WT mice. shRNA-GDPD3 mice were more responsive to both low-force (0.07 g) and high-force (0.4 g), from POD28 to POD45 von Frey fibers had a significantly lower frequency of foot withdrawal than WT mice. From POD28 to POD45, shRNA-GDPD3 mice had higher mechanical and thermal pain thresholds than WT mice (Fig. 4E–G).

**Fig. 5:**
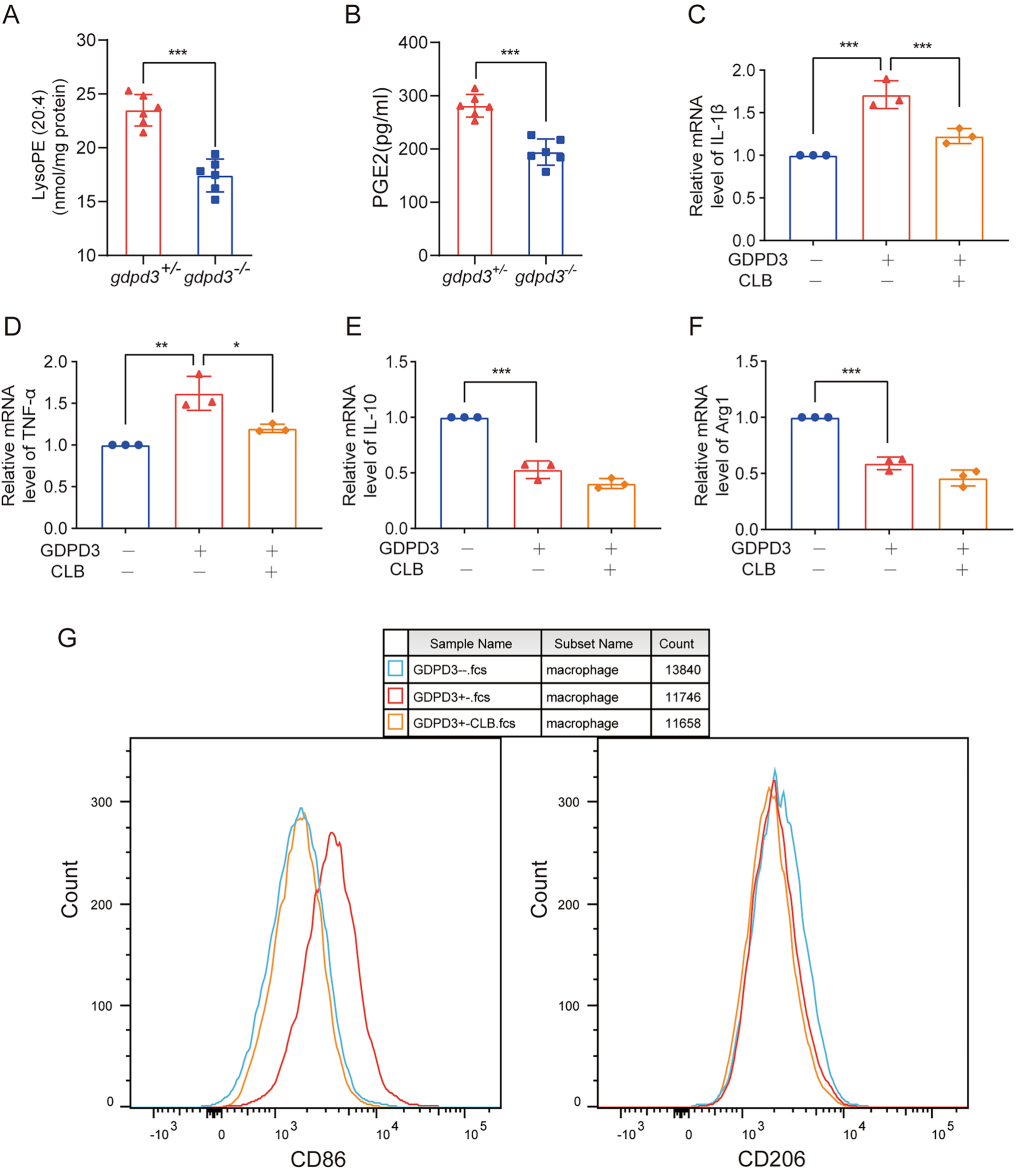
GDPD3 promotes M1 macrophage polarization through PGE2. **A** Mass spectrometry results of phosphatidylethanolamine (PE) (20:4) in GDPD3^−/−^ and GDPD3^+/−^ BMDMs treated with LPS. **B** ELISA results of PGE2 in GDPD3^−/−^ and GDPD3^+/−^ BMDMs treated with LPS. **C**, **D**. PGE2 inhibitor Celecoxib (CLB) reversed the promotion effect of GDPD3 on M1 macrophage polarization-related proinflammatory cytokines (IL-1β and TNF-α) in BMDMs challenged with LPS. **E**, **F** CLB could not reverse the inhibitory effect of GDPD3 on M2 macrophage polarization-related gene IL-10 and Arg1 expression via Q-PCR analysis in BMDMs challenged with IL-4. **G** Flow cytometry results of GDPD3 promoting M1 macrophage polarization via PGE2.The data were presented as mean ± SD, n = 3, *P < 0.05, **P < 0.01, ***P < 0.001

The levels of phosphatidylethanolamine (PE) (20:4) indicating arachidonic acid and prostaglandin E2 (PGE2) in GDPD3^−/−^ BMDMs were significantly lower than those in GDPD3^±^ BMDMs, which have already been reported to mediate inflammatory pain (Fig. 5A, B). Pro-inflammatory cytokines including IL-1βand TNFαincreased relatively inferior in GDPD3^−/−^ BMDMs challenged with LPS to GDPD3^+/−^ BMDMs, indicating that GDPD3 promotes M1 macrophagic polarization. However, PGE2 inhibitor Celecoxib (CLB) reversed the effect of GDPD3 on M1 macrophage polarization-related proinflammatory cytokines in BMDMs.

Furthermore, compared to CLB, GDPD3 deficiency had a stronger effect on alleviating DRG neuroinflammation (Fig. 5C, D). In addition, GAPD3 deficiency increases the anti-inflammatory cytokines (IL-10, Arg1) of BMDMs when challenged with LPS, while CLB had no such effect (Fig. 5E, F). Through flow cytometry analysis, both GDPD3 deficiency and CLB could attenuate M1 polarization of BMDMs under LPS stimulation, while GDPD3 deficiency, but not CLB, could slightly facilitate M2 polarization (Fig. 5G).

### GDPD3 Acts Through PPARγ to Regulate Macrophage Polarization

**Fig. 6:**
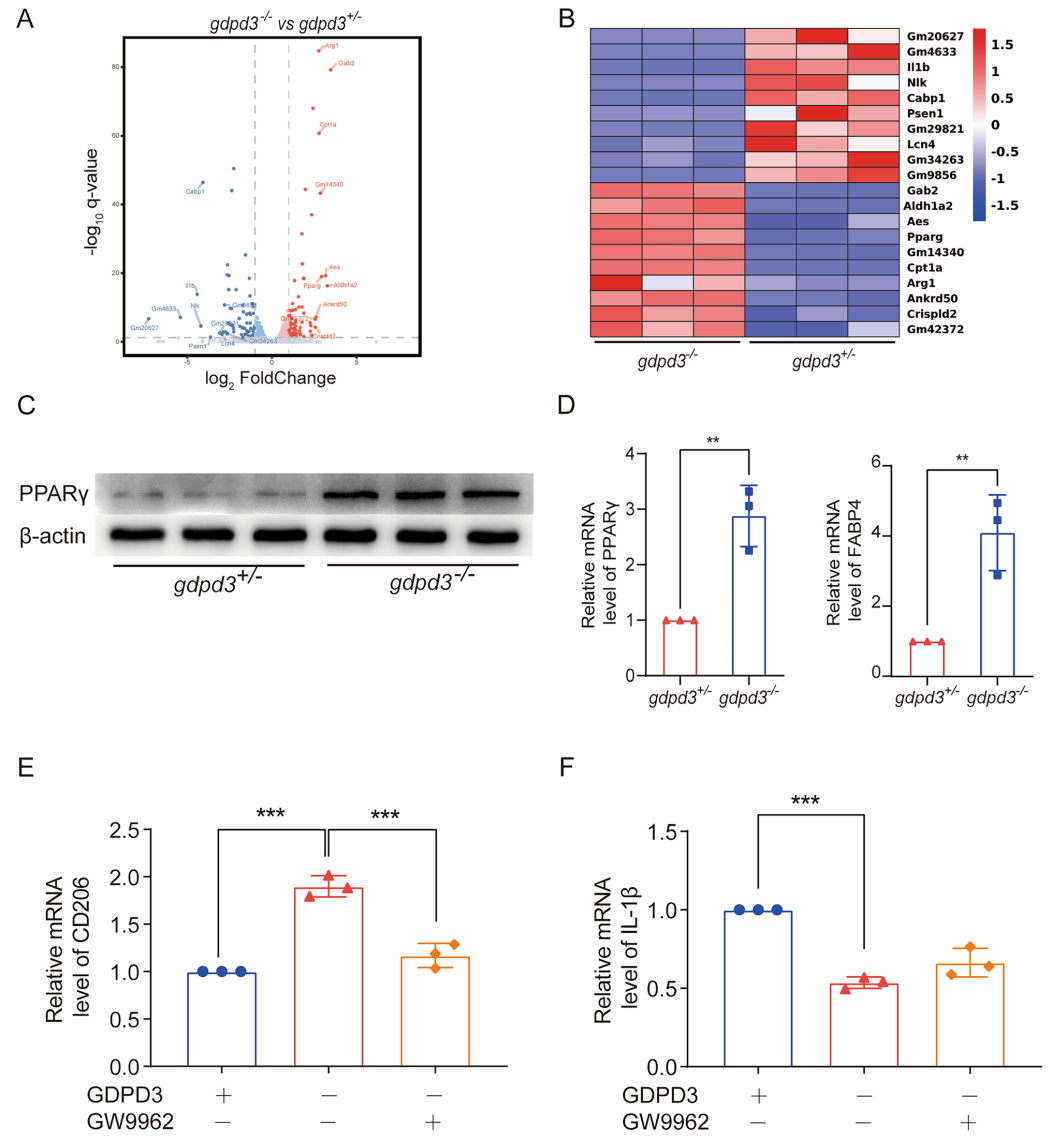
GDPD3 promotes M2 macrophage polarization through PPARγ. Volcano plot **A** and Hierarchical plot **B** show that the overexpressed genes of M2 macrophage polarization in the GDPD3^−/−^ BMDMs were mainly related to the PPARγ-related pathway. PPARγ protein expression **C** and mRNA expression **D** in GDPD3^−/−^ and GDPD3^±^ BMDMs challenged with LPS. **E** FABP4 (downstream of PPARγ) mRNA expression in GDPD3-/- and GDPD3 ± BMDMs challenged with LPS. **F**, **G** PPARγ inhibitor GW9962 reversed the effect of GDPD3 on macrophage polarization-related gene CD206 (M2 polarization) and IL-1β (M1 polarization) expression via RT-QPCR analysis in BMDMs challenged with LPS. The data were presented as mean ± SD, n = 3, **P < 0.01, ***P < 0.001. PPARγ, peroxisome proliferator-activated receptor gamma; FABP4, fatty acid binding protein-4

**Fig. 7:**
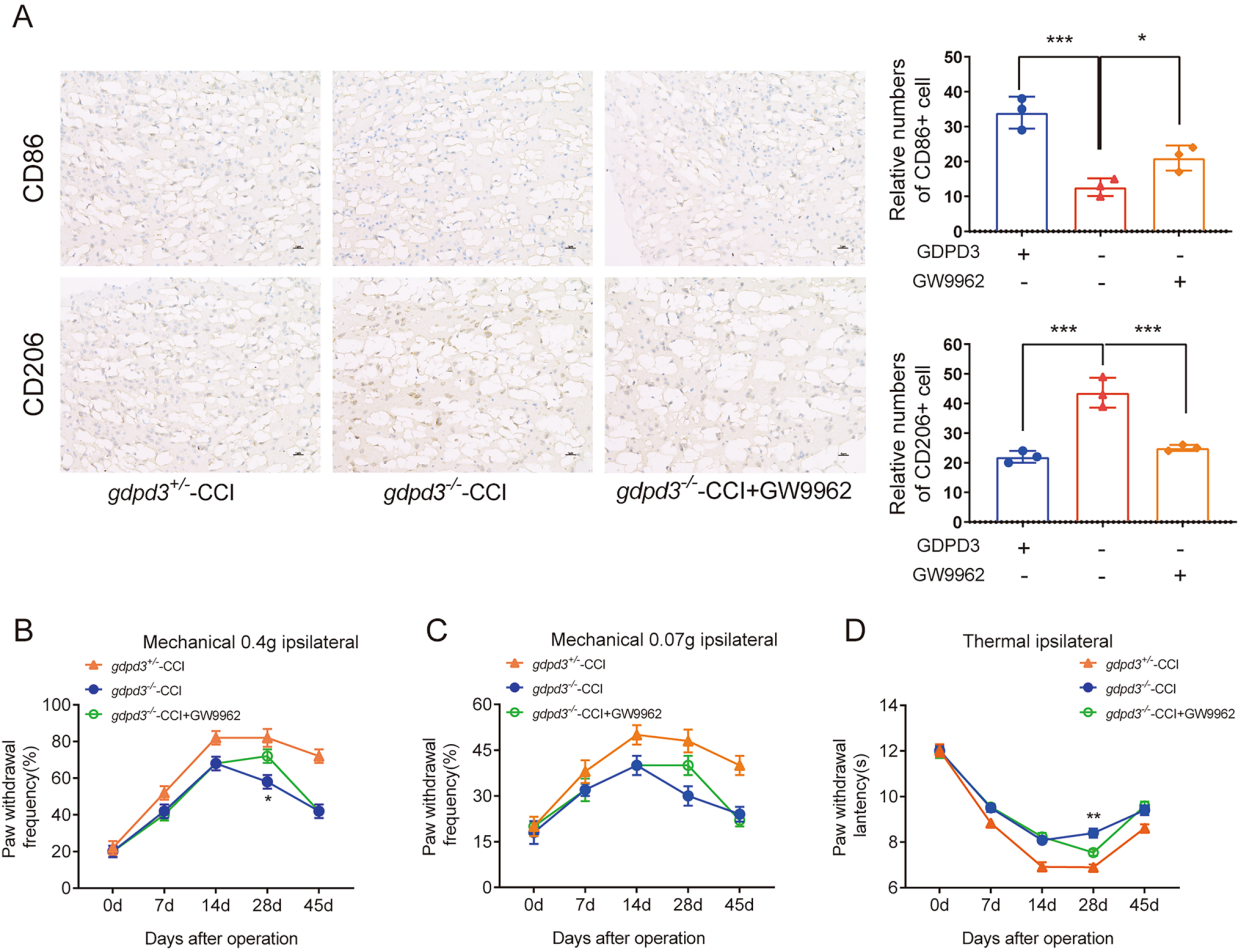
GDPD3 regulates neuropathic pain through PPARγ.A Immunohistochemical analysis of the expression levels of CD86 and CD206 in the DRG of various groups of mice. Mechanical hypersensitivity trend was assessed by the percentage of paw withdrawal to 0.4 g von Frey filament **A** and 0.07 *g* von Frey filament **B** thermal hypersensitivity trend was assessed by paw withdrawal latency to Hargreaves test **C** The data were presented as mean  ±  SD, n = 3, *P < 0.05, **P < 0.01, ***P < 0.001

Through RNA sequencing of GDPD3^−/−^ and GDPD3^+/−^ BMDMs, challenged with LPS, we found that GDPD3 deficiency was correlated with peroxisome-proliferator-activated receptor γ (PPAR γ) and fatty acid binding protein 4 (FABP4), a downstream molecule of PPARγ (Fig. 6A, B). The levels of PPARγ protein and mRNA in GDPD3^−/−^ BMDMs challenged with LPS were higher than those in GDPD3^±^ BMDMs (Fig. 6C, D). The results of RT-QPCR experiments showed that GDPD3 deficiency increased the transcriptional level of CD206 (M2 marker) and decreased the mRNA level of IL-1β (M1 marker) in BMDMs challenged with LPS. However, GW9962 (inhibitor of PPARγ) reversed the effect of GDPD3 on macrophagic polarization, indicating that the modulating effect of GDPD3 deficiency on macrophagic polarization was through PPAR γ pathway (Fig. 6E, F). In addition, the results of immunohistochemistry showed that GDPD3 deficiency increased the protein level of CD206 (M2 marker) and decreased the protein level of CD86 (M1 marker) in BMDMs. However, GW9962 reversed the effect of GDPD3 on macrophagic polarization, indicating that the modulating effect of GDPD3 deficiency on macrophagic polarization was through PPARγ pathway (Fig. 7A). Behavioral tests were conducted to observe the effect of GW9962 on hyperalgesic behavior. The experimental results showed that GDPD3 deficiency in CCI mice resulted in a significant increase in mechanical and thermal pain thresholds at POD28 and POD45 days postoperatively, whereas the results at POD0, 7, and 14 were not significantly altered; based on this, the inhibition of PPAR γ activity using GW9962 reversed the GDPD3 knockdown effect, lowering mechanical and thermal pain thresholds at POD28 without significant changes in behavioral outcomes at POD0, 7 and 14 of modelling, these results suggest that the modulatory effect of GDPD3 deficiency on nociception is mediated through the PPAR γ pathway (Figs. 7B–D).

## Discussion

The development of effective treatments for neuropathic pain remains a significant challenge in the field of pain management. The current study provides compelling evidence that GDPD3 plays a pivotal role in the modulation of macrophage polarization and the pathogenesis of neuropathic pain. The observed deficiency of GDPD3 leading to the alleviation of pain in a CCI model opens a novel avenue for therapeutic strategies targeting neuropathic pain.

In our study, establishing a stable CCI model facilitated the exploration of the complex relationship between macrophage polarization and neuropathic pain. The upregulation of the M1 marker CD86 and the delayed expression of the M2 marker CD206 in CCI mice suggest the pathological role of M1 polarized macrophages in neuropathic pain, consistent with previous studies [11, 12]. M1 macrophages, as pro-inflammatory cells, are closely associated with the progression of various neurological diseases, including neuropathic pain [13, 14], and inhibiting M1 polarization is key for treating neuropathy [15]. Our further results indicate a significant increase in GDPD3 expression in the DRGs of CCI mice, correlating with the peak of M1 polarization, leading us to speculate that GDPD3 may be a potential modulator of inflammation and pain.

In vitro experiments further supported the role of GDPD3 in promoting M1 polarization, with LPS and IL-4 respectively inducing macrophage polarization towards M1 and M2 [16]. The results suggest that GDPD3 deficiency leads to a reduction in pro-inflammatory cytokines associated with M1 and an increase in anti-inflammatory cytokines associated with M2. This polarization shift from M1 to M2 is not only at the cellular level but also corresponds to a significant reduction in mechanical and thermal hypersensitivity responses in GDPD3-deficient mice at the behavioral level, highlighting the functional significance of GDPD3 in pain perception. The ratio of M1/M2 macrophages to neuropathic pain has been well studied [17, 18], but to our knowledge, this is the first report of GDPD3's role in regulating macrophage polarization and neuropathic pain. Interestingly, GDPD3^−/−^ mice in this study only showed significant pain relief on postoperative days 28 and 45, which is inconsistent with most previous studies [19, 20]. This may be because GDPD3 expression levels in CCI mice only began to rise significantly on day 14 post-surgery, peaking on day 28 and maintaining until day 45, resulting in pain relief not being significant from 0 to 14 days post-surgery. Although GDPD3 expression levels rose significantly by day 14, the behavioral manifestations have a certain latency, hence the relief only appeared on day 28. Using GDPD3-deficient mice and shRNA-mediated GDPD3 knockdown in DRGs further emphasized the potential of GDPD3 as a therapeutic target for neuropathic pain. Additionally, knocking out GDPD3 in WT mice did not affect the pain threshold, reflecting to some extent the safety of GDPD3 as a therapeutic approach for neuropathic pain.

Our findings also suggest that the anti-inflammatory and analgesic effects of GDPD3 knockout are superior to the PGE2 inhibitor celecoxib. This indicates that targeting GDPD3 may offer a more comprehensive approach to reprogramming macrophage polarization and alleviating pain compared to targeting downstream mediators like PGE2 alone. Considering the role of PGE2 in inflammatory pain [21], our study suggests that the impact of GDPD3 on macrophage polarization and neuropathic pain is at least partly mediated by regulating PGE2 levels. Moreover, the association of GDPD3 deficiency with increased PPARγ expression and its downstream effector FABP4 provides mechanistic insights into the regulation of macrophage polarization. PPARγ is known for its role in lipid metabolism and inflammation, and its agonists have been shown to have analgesic effects in preclinical pain models [22, 23]. The PPARγ inhibitor GW9962 reversed the effects of GDPD3 deficiency on macrophage polarization and pain behavior, further confirming the key role of PPARγ in the anti-inflammatory and analgesic effects of PDGD3. In this study, we discovered for the first time that GDPD3 can regulate M1 macrophage polarization through the PGE2 pathway and the PPARγ/FABP4 pathway. Previous studies have shown a complex relationship between the PGE2 pathway and the PPARγ/FABP4 pathway, with both PPARγ and PGE2 being downstream of macrophage oxidative phosphorylation, which can regulate M2 polarization [24]. Additionally, exogenous PGE2 can inhibit the expression of PPARγ and FABP4 in mesenchymal stem cells [25], highlighting the importance of PGE2 and PPARγ/FABP4 in inflammatory responses.

Despite these promising findings, our study has limitations that warrant consideration. The use of animal models, while invaluable, may not fully recapitulate the human condition of neuropathic pain. Additionally, the mechanisms by which GDPD3 influences macrophage polarization and pain are likely to be multifaceted and require further elucidation. The downstream signaling pathways and potential off-target effects of GDPD3 deficiency also remain to be fully explored.

In summary, GDPD3 regulates macrophagic polarization and neuroinflammation in neuropathic pain through PGE2 and PPARγ/FABP4 pathway. Specifically, GDPD3^-/-^ mice showed an elevating tolerance in pain, indicating that GDPD3 deficiency can alleviate symptoms of neuropathic pain. The GDPD3^-/-^ BMDM tends to M2 polarization instead of M1 polarization. The regulation of GDPD3 on M1 polarization is closely related to PGE2, while the regulation of GDPD3 on M2 polarization is mediated throUGH PPAR Γ/ FABP4 PATHWAY.

### Supplementary Information

Below is the link to the electronic supplementary material.

Supplementary file1 (TIF 341 kb)

Supplementary file2 (DOCX 22 kb)

## Data Availability

The datasets used and/or analyzed during the current study are available from the corresponding author on reasonable request.
