# Supplementary figures and images for: GDPD3 Deficiency Alleviates Neuropathic Pain and Reprograms Macrophagic Polarization Through PGE2 and PPARγ Pathway
Source: Neurochem Res. 2024 May 20;49(8):1980–92. doi: 10.1007/s11064-024-04148-2 (PMC11233315; doi:10.1007/s11064-024-04148-2)

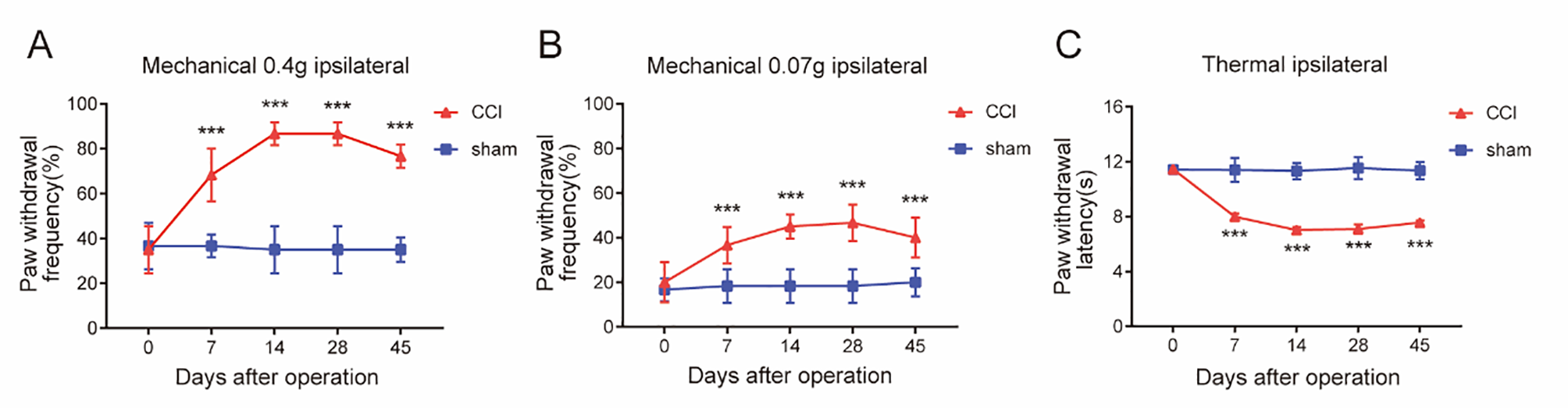

Supplement: Supplementary file 1 — Supplementary file1 (TIF 341 kb) [file 11064_2024_4148_MOESM1_ESM.tif]
