# Supplementary material for: GDPD3 Deficiency Alleviates Neuropathic Pain and Reprograms Macrophagic Polarization Through PGE2 and PPARγ Pathway
Source: Neurochem Res. 2024 May 20;49(8):1980–92. doi: 10.1007/s11064-024-04148-2 (PMC11233315; doi:10.1007/s11064-024-04148-2)
Supplement: Supplementary file 2 — Supplementary file2 (DOCX 22 kb) [file 11064_2024_4148_MOESM2_ESM.docx]

**Table S1 RT-qPCR Primer sequence**

| mRNA | Sample |  |
| --- | --- | --- |
| GDPD3 | F: 5'- AGGCTATGATCCCTCTCCTGTACT -3'  R: 5'- CCTCTAGGCCCCAAGACTCT -3' |  |
| PPARγ | F: 5'- GGAAGACCACTCGCATTCCTT -3'  R: 5'- GTAATCAGCAACCATTGGGTCA -3' |  |
| FABP4 | F: 5'- AAGGTGAAGAGCATCATAACCCT -3'  R: 5'- TCACGCCTTTCATAACACATTCC -3' |  |
| IL-1b | F: 5'- ATGGCAGAAGTACCTAAGCTC -3'  R: 5'- TTAGGAAGACACAAATTGCATGGTGAACTCAGT -3' |  |
| TNF-a | F: 5'- ATGAGCACTGAAAGCATGATC -3'  R: 5'- TCACAGGGCAATGATCCCAAAGTAGACCTGCCC -3' |  |
| IL-10 | F: 5'- AGGGCACCCAGTCTGAGAACA -3'  R: 5'- CGGCCTTGCTCTTGTTTTCAC -3' |  |
| Arg1 | F: 5'- CATTGGCTTGCGAGACGTAGAC -3'  R: 5'- GCTGAAGGTCTCTTCCATCACC -3' |  |
| GAPDH | F: 5'-GACAGTCAGCCGCATCTTCT-3'  R: 5'-GCGCCCAATACGACCAAATC-3' |  |

Note: F: Forward；R: Reverse
